# Anticolon Cancer Targets and Molecular Mechanisms of Tao-He-Cheng-Qi Formula

**DOI:** 10.1155/2022/7998664

**Published:** 2022-04-18

**Authors:** Zexin Zhang, Siqi Lin, Zifeng Liu, Jun Han, Jing Li, Yi Yu

**Affiliations:** ^1^The First Clinical Medical College of Guangzhou University of Chinese Medicine, Guangzhou 510405, China; ^2^The Second Clinical Medical College of Guangzhou University of Chinese Medicine, Guangzhou 510405, China; ^3^National and Local Joint Engineering Research Center for Key Technology of Chinese Medicinal Composition Granules, Beijing Tcmages Pharmaceutical Co., Ltd., Beijing 101301, China; ^4^The First Affiliated Hospital of Hunan University of Chinese Medicine, Changsha 410000, China

## Abstract

**Background:**

Tao-He-Cheng-Qi Formula (THCQF) is a traditional Chinese medicine that has been proven to have antitumor effects. The aim of this study was to elucidate the molecular targets and mechanisms of THCQF against colon cancer and construct a prognostic model based on network pharmacology, bioinformatics analysis, and in vitro experiments.

**Methods:**

Potential THCQF compounds and targets were retrieved from the Traditional Chinese Medicine Systems Pharmacology and Bioinformatics Analysis Tool for Molecular Mechanism of Traditional Chinese Medicine databases. Differentially expressed genes for colon cancer were screened in The Cancer Genome Atlas and Gene Expression Omnibus databases. The anticolon cancer mechanisms of THCQF were explored using Gene Ontology (GO) and Kyoto Encyclopedia of Genes and Genomes (KEGG) enrichment analyses. Molecular docking simulations and molecular dynamics analysis were used to evaluate the binding between target proteins and active compounds. Finally, the identified compounds were used to treat colon cancer cells from the HCT116 cell line, and expression of mRNA and protein after relevant posttreatment were tested using real-time polymerase chain reaction and western blotting.

**Results:**

A total of 27 anticolon cancer targets of THCQF were selected, among which four genes (*CCNB1*, *CCNA2*, *IL1A*, and *MMP3*) were shown to effectively predict patient outcomes in a prognostic colon cancer model. GO and KEGG enrichment analyses indicated that the activity against colon cancer of THCQF was associated with the interleukin (IL)-4 and IL-3 signaling pathways. Two compounds in THCQF, aloe emodin (AE) and quercetin (QR), were shown to efficiently bind to cyclin B1, the protein encoded by *CCNB1*. Finally, incubation of HCT116 cells with AE and QR significantly decreased *CCNB1* mRNA expression and cyclin B1 levels.

**Conclusions:**

Taken together, the results indicate that AE and QR are the pivotal active compounds of THCQF, and *CCNB1* is the main molecular target through which THCQF exerts its anticolon cancer effects. The study findings provide insight for studies investigating the anticancer effects of other traditional Chinese medicines.

## 1. Background

Colon cancer is a ubiquitous gastrointestinal malignancy that accounts for 6% of new cancer cases and 5.8% of cancer deaths annually in the world [1]. The incidence of colon cancer has increased yearly owing to improved economic standards, which have altered the diet structure and habits of many populations [2]. Patients with early-stage colon cancer may not exhibit obvious symptoms, but they gradually develop altered bowel habits, fecal characteristics, and abdominal pain [3]. By the time patients with colon cancer seek treatment, 25% of cases experience tumor metastasis, which results in poor treatment outcomes [4]. Despite advances in colon cancer treatment, including surgery, chemotherapy, and radiotherapy [5], the survival rate of 5-year colon cancer patients with stage IV remains less than 12% [6, 7]. Therefore, more effective therapeutic approaches for colon cancer are urgently needed.

Tao-He-Cheng-Qi Formula (THCQF) is a Traditional Chinese Medicine (TCM) that originates from the “*Treatise on Cold Damage Diseases*,” an ancient medical textbook compiled by Zhang Zhongjing. THCQF is composed of the following five herbs and minerals: *Rheum palmatum* L. [Chinese rhubarb, dahuang (DH)], *Prunus persica* (L.) Batsch [peach kernel, taoren (TR)], mangxiao (Glauber's salt, MX), *Neolitsea cassia* (L.) Kosterm. [cassia twig, guizhi (GZ)], and *Glycyrrhiza glabra* L. [licorice, gancao (GC)] [8]. The formulation contains 12 g TR, 12 g DH, 6 g GZ, 12 g GC, and 6 g MX. THCQF has been used in East Asia to treat interior heat patterns and resolve blood stasis for more than a thousand years [9]. It has also been applied to treat acute liver failure [10], chronic kidney disease [11], and intracerebral hemorrhage [12]. Most importantly, THCQF has an inhibitory effect against cancers [13]. For example, THCQF inhibited the expression of *BCL-2* and *PCNA* to suppress cancer development [14]. Furthermore, THCQF enhanced human immunity against cancer by promoting *TNF-α* expression [15]. Previous studies have shown that THCQF treatment can help alleviate the discomfort of patients with colon cancer [16] and prevent disease recurrence [17]. Nevertheless, the molecular targets and mechanisms of THCQF against colon cancer remain unclear and warrant further elucidation.

Tumor cells are characterized by various genetic changes and chromosomal instabilities that can be identified by bioinformatics analysis, which may enable a personalized approach to cancer treatment and help identify new drugs that target specific proteins [18]. The network pharmacology approach, which reveals drug-gene-disease comodule associations, has been widely used to predict target profiles and pharmacological effects of herbal compounds [19]. However, the effectiveness of predicted targets needs to be verified. Therefore, in addition to exploring the anticolon cancer molecular targets and mechanisms of THCQF using network pharmacology and bioinformatics analysis, our study aimed to construct a prognostic model of survival status and validate the effects of the identified active compounds on the predicted targets in colon cancer cells.

## 2. Methods

### 2.1. Screening of THCQF Candidate Compounds and Targets

**Figure 1:**
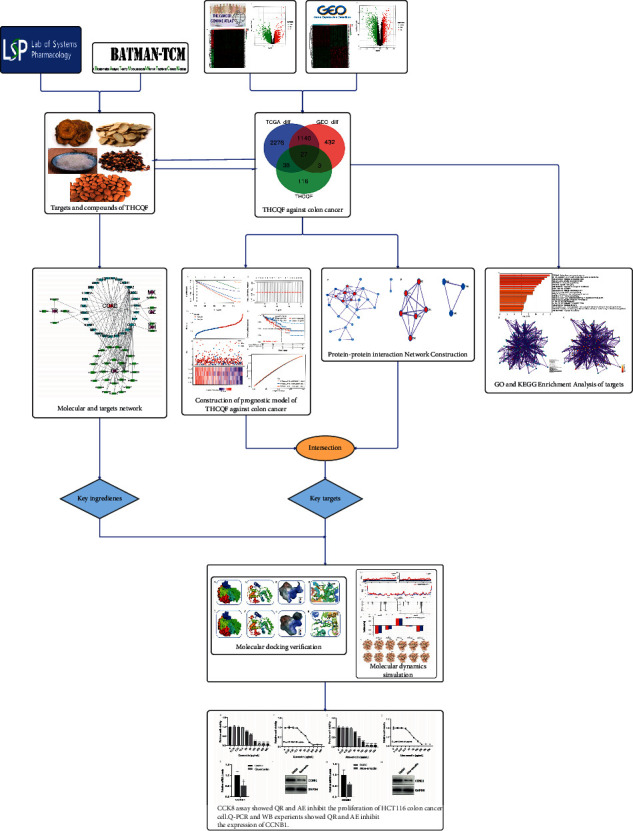
Flowchart of the study progression.

The workflow of this study was shown in Figure 1. The major candidate compounds of THCQF were obtained from the TCM systems pharmacology (TCMSP) database (https://lsp.nwu.edu.cn/tcmsp.php) using the following criteria: oral bioavailability ≥30% and drug likeness ≥0.18. In the TCMSP database, relationships between Chinese herbal medicines, targets, and diseases are captured. Chemicals, targets, and drug-target networks are included, along with pharmacokinetic properties such as oral bioavailability, drug likeness, intestinal epithelial permeability, blood-brain barrier in this database [20]. Because the TCMSP database lacks information on minerals, the major compounds of MX were screened in the Bioinformatics Analysis Tool for Molecular Mechanism of TCM (BATMAN-TCM) database (https://bionet.ncpsb.org/batman-tcm/) using the following criteria: score cutoff ≥20 and adjusted *P* value < 0.05. The BATMAN-TCM database enables predictions of potential targets for each query ingredient, and performs Gene Ontology (GO) term and Kyoto Encyclopedia of Genes and Genomes (KEGG) pathway enrichment analyses for target compounds. These functions contribute to the understanding of multicomponent, multitarget, and multipathway therapeutic mechanisms of TCMs and provide clues for further experimental validation [21]. Perl software was performed to create a candidate compound and target network.

### 2.2. Identification of Therapeutic Targets of THCQF against Colon Cancer

Transcriptome data for colon cancer and associated metadata were downloaded from The Cancer Genome Atlas (TCGA) database (https://portal.gdc.cancer.gov/). Gene expression profiles from GSE68468, a colon cancer dataset, and GPL96, the Affymetrix Human Genome U133A Array dataset, were downloaded from the Gene Expression Omnibus (GEO) database (https://www.ncbi.nlm.nih.gov/geo/). The “limma” package in R software was applied to detect differentially expressed genes (DEGs) between normal and tumor groups using the following criteria: log∣fold change∣ > 1, adjusted *P* value <0.05, respectively. The THCQF targets were integrated with the DEGs to identify therapeutic targets of THCQF against colon cancer.

### 2.3. Construction of a Prognostic Model of Colon Cancer

Univariate Cox regression analysis was employed to screen for anticolon cancer targets of THCQF that were significantly associated with patient survival. Least absolute shrinkage and selection operator (LASSO) regression analysis was used to remove redundant factors and build a prognostic model. The LASSO-Cox model has been widely applied for cancer survival prediction and detecting associated genes because it is capable to identify these genes that with high internal consistency [22, 23]. Kaplan–Meier (KM) survival analysis was used to divide the risk scores of the model into high- and low-risk groups, with a *P* value <0.05 in the log-rank test considered to be significant. In addition, receiver operating characteristic curves were adopted to evaluate the predictive capability of the model for 1-, 3-, and 5-year survival. An area under the curve (AUC) > 0.5 was considered to indicate good predictive capability.

### 2.4. Construction of a Protein-Protein Interaction (PPI) Network and Identification of Key Targets

To identify the hub target genes of THCQF against colon cancer, a PPI network was constructed using Metascape (https://metascape.org/gp/index.html). Targets in the network were clustered based on their interaction relationships, and those with similar functions were grouped into modules. The genes selected for the prognostic model were overlapped with the targets of the PPI network to identify the hub targets that THCQF anticolon cancer.

### 2.5. Prediction of Candidate Anticolon Cancer Compounds Based on the Molecule-Target Network Construction

To identify the pivotal candidate compounds of THCQF with anticolon cancer activity, a molecule-target network was built up using Cytoscape version 3.7.2 software. The THCQF-derived compounds that targeted the hub patient survival-associated genes were considered core candidates for further research.

### 2.6. GO and KEGG Functional Enrichment Analyses of Therapeutic Targets of THCQF against Colon Cancer

GO and KEGG functional enrichment analyses of the therapeutic targets of THCQF against colon cancer were performed using Metascape according to the criteria: *P* value <0.01 and minimum enrichment >1.5. Additionally, an interaction network was constructed to explore possible connections between the identified biological functions and pathways.

### 2.7. Molecular Docking Simulation

Molecular docking simulations were performed to verify the binding abilities of the active small molecules to the identified proteins. The three-dimensional protein structures were downloaded from the Protein Data Bank (https://www.rcsb.org/). Subsequently, hydrogens, charges, and protons were added to the proteins using the optimized potentials for liquid simulation (OPLS) 2005 force field and energy optimization was carried out using the “Protein and Ligand Preparation” module in Schrödinger software. The “Glide” docking method in Schrödinger software was used to predict the binding modes of the small molecules and proteins.

### 2.8. Molecular Dynamics Analysis

The stability of the predicted small molecule-protein complexes was assessed using Amber molecular dynamics v14 software. The small molecule-protein complexes predicted via molecular docking simulation were used as the initial structures. The general Amber force field (GAFF) was applied for ligands, and Amber ff14SB force field was used for proteins. The default protonation state was used for amino acid residues of the proteins, and hydrogenation was carried out using the “tleap” module. The optimized structures and electrostatic potential of the small molecule-protein complexes were calculated using the “Gaussian09” quantum mechanical software package with the density functional theory level B3LYP/6-31G. The partial charges of the small molecules were obtained using the restrained electrostatic potential method in the “Antechamber” module. A constant volume was used to heat the system from 0 to 300 K within 60 picoseconds (ps), and the solvent density was balanced in a constant temperature-pressure system (*T* = 300 K, *P* = 1 atm). Sampling was set to 100 nanoseconds under constant pressure conditions with one frame per ps for subsequent analysis of conformation. The stability of the protein was evaluated using the root-mean-square deviation (RMSD), while the flexible region of the protein was assessed by the root-mean-square fluctuation (RMSF).

### 2.9. Cell Viability Assay

To verify the anticolon cancer activity of the identified compounds, HCT116 colon cancer cells, which were purchased from the Chinese Academy of Sciences Cell Bank and routinely cultured in DMEM/F12 medium containing 10% fetal bovine serum in a saturated humidity incubator with 5% CO_2_ at 37°C, were incubated with different concentrations of candidate compounds for 72 h, and the Cell Counting Kit-8 assay was performed to assess cell viability. The half-maximal inhibitory concentration (IC_50_) of each compound against HCT116 cells was calculated. Subsequently, HCT116 cells were incubated with the IC_50_ of each compound for 12 h, and the relevant mRNA and protein expression levels were evaluated using real-time polymerase chain reaction (RT-PCR) and western blotting, respectively. Aloe emodin (AE; Lot 210716, purity >98%) and quercetin (QR; Lot 210621, purity >98%) were obtained from Chengdu PureChem-Standard Co., Ltd. (Chengdu, China).

### 2.10. RT-PCR

Reverse transcription was carried out using an RT-PCR kit (Novizan Biotechnology Co., Ltd., Nanjing, China) according to the manufacturer's instructions with 1 *μ*g total RNA. The reaction was prepared as shown in Table 1. After reverse transcription, the reaction mixture was diluted 1 : 10 with ddH_2_O and stored at −20°C. Subsequently, RT-PCR was carried out in a 96-well plate using a real-time fluorescence quantitative PCR instrument (CFX96 Bio-Rad) according to the manufacturer's instructions. The specific primers are listed in Table 2, and *GAPDH* was used as the internal reference for normalization of gene expression. The 2^−ΔΔCt^ method was used for relative quantification of gene expression. Each compound was tested in triplicate.

### 2.11. Western Blotting

Samples with equal amounts of protein, along with molecular weight standards, were separated by sodium dodecyl sulfate-polyacrylamide gel electrophoresis at 100 V for 1-2 h. The separated proteins were transferred to a polyvinylidene fluoride membrane at a constant current of 200 mA for 1 h. The membrane was incubated with 5% blocking buffer at room temperature for 1 h, followed by incubation with a primary antibody with a concentration 1 : 1000 at an appropriate dilution at room temperature for an additional 2 h. After that, the membrane was washed 3 times successively with TBST (Tris-buffered saline with Tween 20) for 5 minutes. Subsequently, the membrane was incubated for 1 h at room temperature in TBST containing 5% blocking reagent and a labeled secondary antibody with a concentration 1 : 5000 and then washed three times with TBST for 5 min each. Finally, the membrane was treated with an enhanced chemiluminescence reagent to visualize the protein bands. GAPDH was used as the internal reference for protein expression, and the relative protein expression in each band was quantified using Quantity One software (Bio-Rad Laboratories, Hercules, CA, USA).

## 3. Results

### 3.1. Identification of THCQF Candidate Compounds and Therapeutic Targets against Colon Cancer

The candidate THCQF compounds identified in the TCMSP and BATMAN-TCM databases included 16 compounds from DH, 92 from GC, 7 from GZ, 1 from MX, and 23 from TR. A total of 184 targets of THCQF were obtained by mapping the candidate ingredients and potential genes.

**Figure 2:**
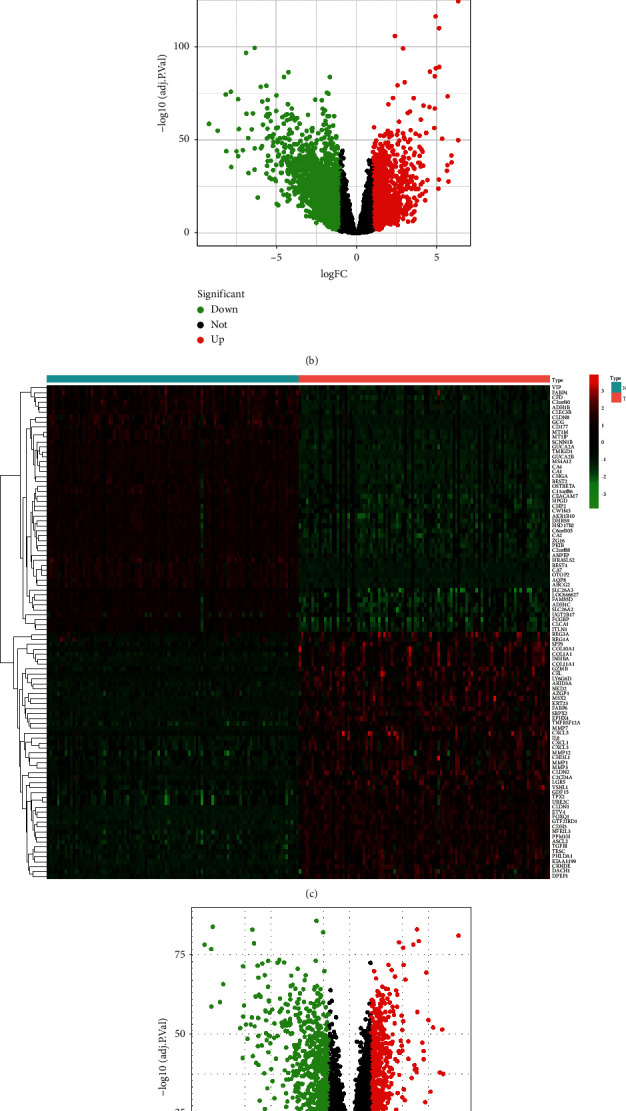
Identification of therapeutic targets of THCQF against colon cancer. (a, b) Differentially expressed genes (DEGs) in colon cancer obtained from TCGA. (c, d) DEGs in the GSE68468 dataset. (e) Integrating the targets of THCQF, DEGs from TCGA, and DEGs in the GSE68468 dataset.

There were 3,481 and 1,602 DEGs were screened out in the TCGA database (Figures 2(a) and 2(b)) and GSE68468 dataset (Figures 2(c) and 2(d)), respectively. By integrating the THCQF targets with the DEGs, 27 therapeutic targets of THCQF against colon cancer were finally identified (Figure 2(e)).

### 3.2. Construction of a Prognostic Model of Colon Cancer

**Figure 3:**
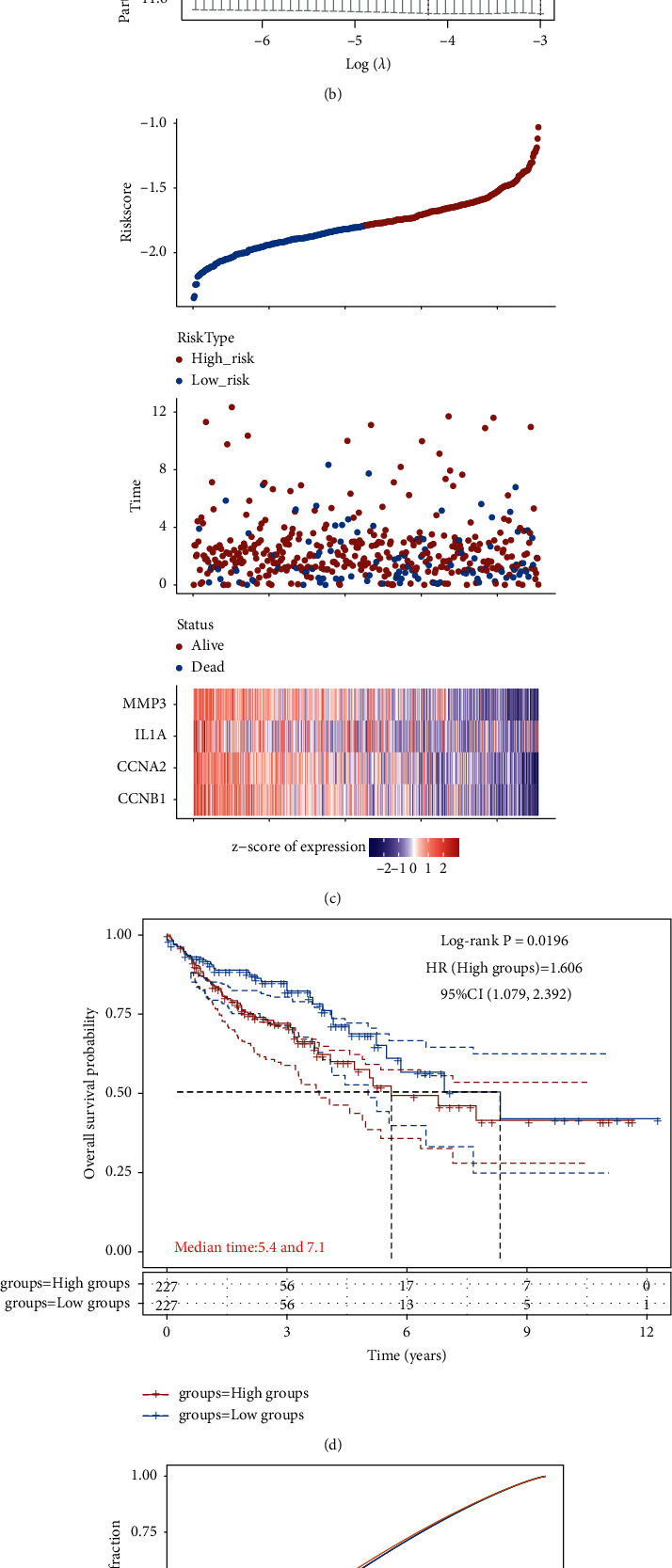
Construction of prognostic model of THCQF against colon cancer. (a, b) LASSO regression analysis removed redundant factors. (c) Risk scores of patients in high- (red) and low- (blue) risk groups. (d) KM survival analysis of patients divided into two groups based on risk. (e) ROC curve of prognostic model.

Univariate Cox regression analysis identified four genes (*CCNB1*, *CCNA2*, *IL1A*, and *MMP3*) that significantly affected patient survival based on the 27 anticolon cancer targets of THCQF. Redundant factors were removed by LASSO regression analysis and *CCNB1*, *CCNA2*, *IL1A*, and *MMP3* were used to build the prognostic model (Figures 3(a) and 3(b)). KM survival analysis revealed a significant difference between high- and low-risk groups (*P*=0.0196 in the log-rank test) (Figure 3(d)). The median survival time was 5.4 years in the high-risk group and 7.1 years in the low-risk group. Furthermore, the AUC values were 0.566, 0.575, and 0.581 for 1-, 3-, and 5-year survival, respectively, suggesting that the prognostic model effectively predicted the survival of patients with colon cancer (Figure 3(e)).

### 3.3. Construction of PPI and Molecule-Target Networks and Identification of Key Targets

**Figure 4:**
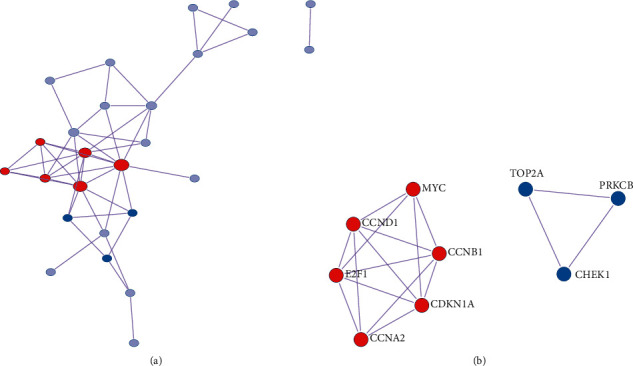
Construction of protein-protein interaction network and filtering of key targets. (a) PPI network construction. (b) Key targets screened by cluster analysis.

The PPI network was built up using 27 therapeutic targets of THCQF against colon cancer (Figure 4(a)). Cluster analysis finally identified two key functional modules with nine therapeutic targets, including *CCNB1*, *CCNA2*, *MYC*, *E2F1*, *CDKN1A*, *CCND1*, *CHEK1*, *TOP2A*, and *PRKCB* (Figure 4(b)). Overlapping the four genes used in the prognostic model with the nine targets of the PPI network yielded two key anticolon cancer targets, namely, *CCNB1* and *CCNA2*.

**Figure 5:**
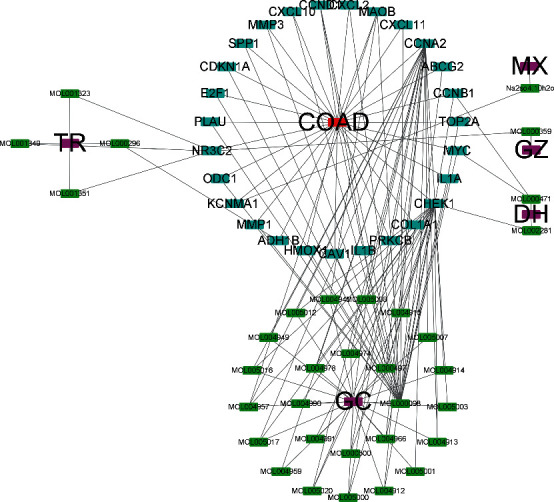
Prediction of candidate compounds with activity against colon cancer based on molecular and target networks.

A molecule-target network was developed to screen out the pivotal candidate ingredients of THCQF with anticolon cancer activity using the 139 compounds and 27 therapeutic targets of THCQF (Figure 5). Within the network, two compounds from DH, four from TR, one from MX, one from GZ, and twenty-five from GC were identified to potentially possess anticolon cancer activity (Table 3). Notably, *CCNB1* was targeted by one compound each from DH and GC, while *CCNA2* was targeted by twenty compounds from GC.

### 3.4. GO and KEGG Functional Enrichment Analyses

**Figure 6:**
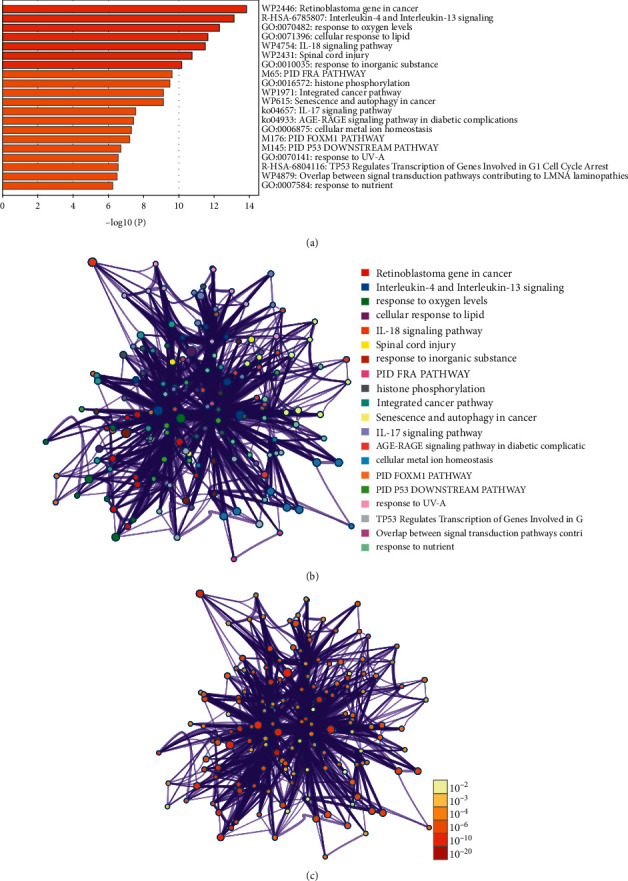
GO and KEGG functional enrichment analyses of therapeutic targets of THCQF against colon cancer.

GO enrichment analysis was employed to examine the functions of biological systems of the 27 therapeutic targets. The results indicated that these genes were involved in oxygen level response and lipid metabolism. In the meantime, KEGG pathway enrichment indicated that the major mechanisms of THCQF inhibited colon cancer were associated with the interleukin (IL)-4 and IL-3 signaling pathways (Figure 6).

### 3.5. Molecular Docking and Molecular Dynamics of Target Proteins and Candidate Compounds

**Figure 7:**
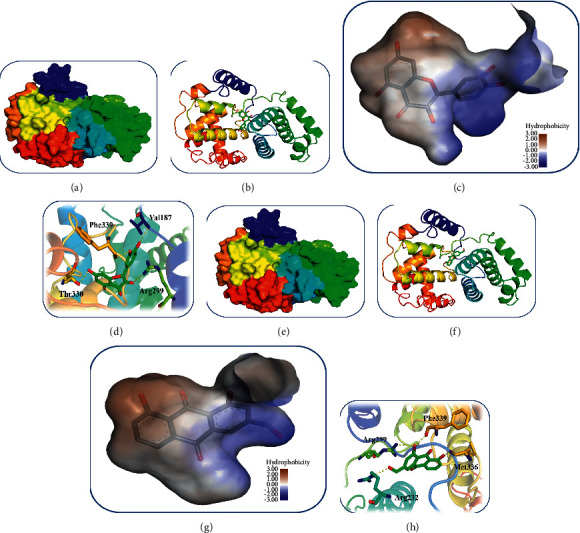
Molecular docking simulations. (a, b) Position of QR bound to cyclin B1. (c) Fragment-based binding of QR with cyclin B1. (d) Mutual effects of QR and cyclin B1. (e, f) Position of AE bound to cyclin B1. (g) Fragment-based binding of cyclin B1 with AE. (h) Mutual effects of AE and cyclin B1.

The molecular docking results indicated that the binding affinity of AE and QR to cyclin B1 was good, the protein encoded by *CCNB1*, compared to other compound-protein complexes (Table 4). Notably, AE had the top-ranked docking score of −6.124 with cyclin B1. Therefore, AE and QR were selected for further investigation (Figure 7).

**Figure 8:**
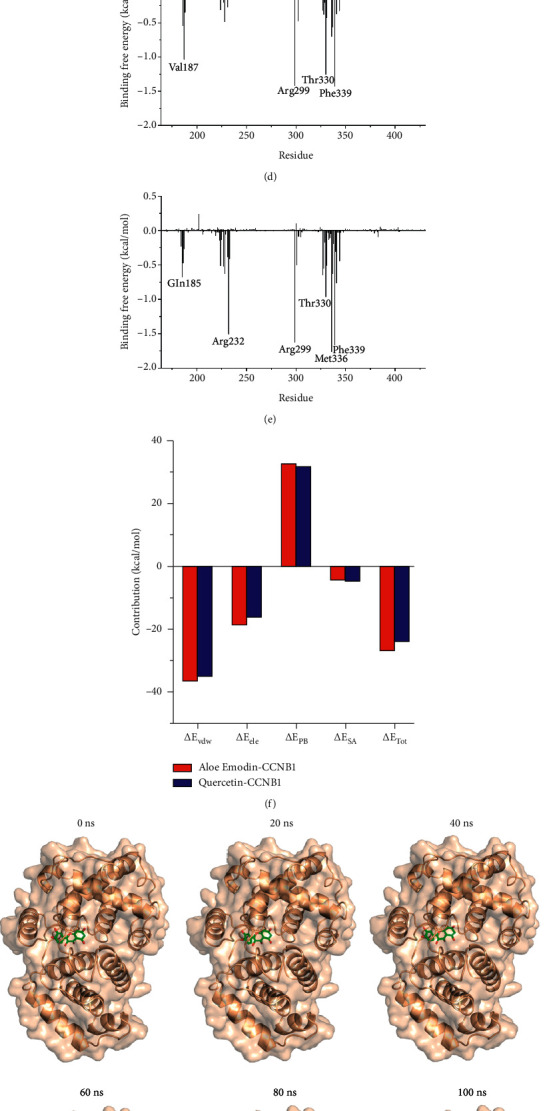
Molecular dynamics analysis. (a, b) RMSD values of QR and AE bound to cyclin B1. (c) RMSF values of QR and AE bound to cyclin B1. (d, e) Calculation of binding free energy of cyclin B1 with QR and AE based on MM/PBSA. (f) Energy decomposition of amino acid residues based on MM/PBSA. (g, h) Motion trails of cyclin B1 bound to QR and AE at different times.

The molecular dynamics results indicated that although the RMSD trajectories of AE and QR bound to cyclin B1 displayed small fluctuations, they remained stable overall. The RMSD values suggested that AE and cyclin B1 formed a more stable complex than QR and cyclin B1, indicating a higher binding affinity between AE and cyclin B1 (Figures 8(a) and 8(b)). Meanwhile, the RMSF values of the AE-bound amino acid residues of cyclin B1 did not significantly fluctuate during the simulation process, indicating that the protein structure was more stable than that formed with QR (Figure 8(c)). The free energy of binding was calculated based on the conformation obtained by the molecular dynamics simulation based on the molecular mechanics/Poisson–Boltzmann surface area method. The total binding free energy (ΔGtot) of AE and cyclin B1 (−26.87 kcal/mol) was higher than that of QR and cyclin B1 (−24.05 kcal/mol), suggesting better binding between AE and cyclin B1 (Figures 8(d)–8(f)). The motion trails of QR and AE bound to cyclin B1 indicated stable binding between these small molecules and the protein (Figures 8(g) and 8(h)).

### 3.6. Validation of Anticolon Cancer Activity of AE and QR

**Figure 9:**
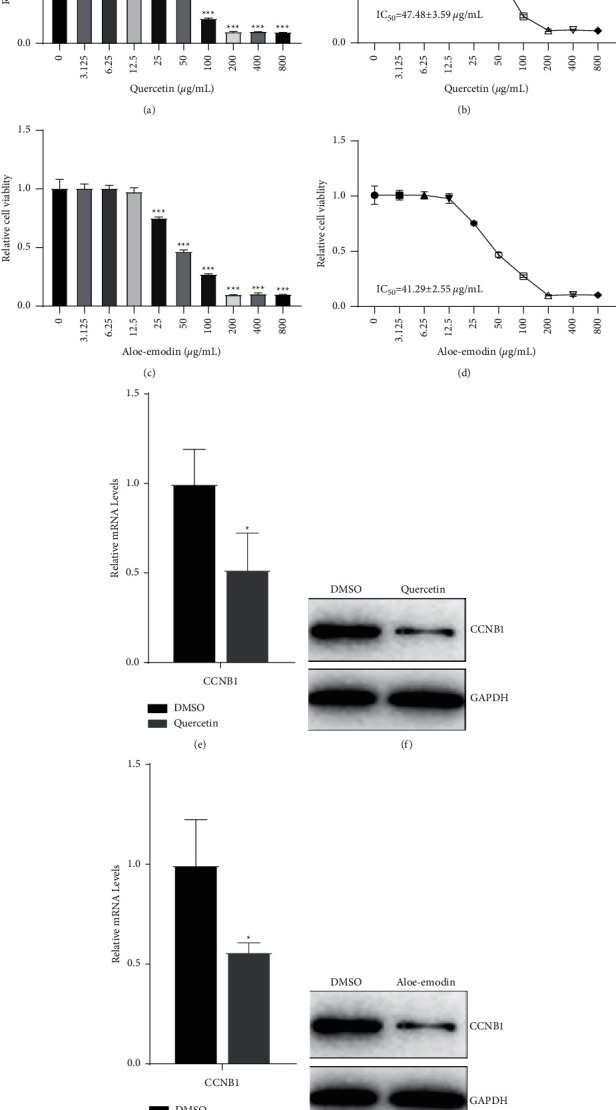
In vitro verification of molecular targets of THCQF against colon cancer. (a, b) Viability of HCT116 cells treated with different concentrations of QR. (c, d) Viability of HCT116 cells treated with different concentrations of AE. (e)-(f) RT-PCR and western blotting results of HCT116 cells treated with IC_50_ of QR. (g, h) RT-PCR and western blotting results of HCT116 cells treated with IC_50_ of AE.

Colon cancer cell growth was not inhibited by AE and QR at concentrations below 25 *μ*g/mL. However, cell growth was significantly inhibited in a concentration-dependent manner at compound concentrations between 25 and 800 *μ*g/mL (Figures 9(a) and 9(c)). Notably, AE had a lower IC_50_ than QR (41.29 ± 2.55 vs. 47.48 ± 3.59 *μ*g/mL, resp.), which concurred with the molecular docking simulation and molecular dynamics analysis results (Figures 9(b) and 9(d)).

To investigate the molecular mechanisms of AE and QR against colon cancer, HCT116 cells were treated with AE and QR at their IC_50_ values, and *CCNB1* expression was examined. Both *CCNB1* mRNA expression and cyclin B1 levels were significantly decreased after AE and QR treatment, further confirming that these two THCQF compounds targeted *CCNB1* to exert anticolon cancer effects (Figures 9(e)–9(h)).

## 4. Discussion

Colon cancer is a common gastrointestinal malignancy with high incidence and mortality, posing a heavy socioeconomic burden [24]. Presently, colon cancer is treated primarily through surgical resection, in combination with chemotherapy and radiotherapy. However, adverse reactions caused by these treatments seriously affect patient quality of life [25]. THCQF is a frequently used TCM against lower energizer heat patterns and blood stasis [26] and is often used as an adjuvant therapy in clinical practice owing to its significant antitumor effects [27]. Emodin, derived from DH, has been confirmed to suppress the growth and invasive ability of SW480 colon cancer cells via the mitogen-activated protein kinase signaling pathway [28]. Aqueous GZ extract exerts an anticancer effect by targeting tubulin proteins, thus impeding mitosis and promoting cell apoptosis [29]. Research has demonstrated that the active compounds of GC may inhibit the proliferation and migration of colorectal cancer cells by blocking the S phase of the cell cycle [30]. Moreover, the application of THCQF has significantly reduced postoperative inflammation, the occurrence of adverse reactions, and restored gastrointestinal function after colon cancer surgery [16]. However, the molecular targets, mechanisms of action, and active ingredients in THCQF have not yet been elucidated.

In the current study, 27 therapeutic targets of THCQF against colon cancer were obtained across multiple databases, and GO and KEGG functional enrichment analyses suggested that the effects of anticolon cancer was closely associated with biological processes such as oxygen level response and lipid metabolism, as well as the IL-4 and IL-3 signaling pathways. IL-4, a multifunctional cytokine produced by T helper 2 cells, is involved in regulation of the cell cycle, cell differentiation, and migration, and has an important effect on immune function [31, 32]. In the early stage of tumor formation, an abnormal immune response caused by IL-4 promotes cancer cell mutation and DNA damage, thus promoting the occurrence and development of tumors [33]. Additionally, the secretion of IL-4 influences the tumor microenvironment by activating M2 macrophages, which leads to tumor immune escape, invasion, and metastasis [34]. IL-3, one of the most common inflammatory growth factors, can stimulate the proliferation and differentiation of myeloid elements [35], activate basophils to participate in the immune response [36], and inhibit angiogenesis by increasing TGF activity early in tumor formation [37]. Furthermore, induction of IL-3 expression in the bone marrow microenvironment of patients with myeloma has been shown to increase bone destruction and promote tumor cell growth [38]. The above results support that the anticancer effects of THCQF may be mediated via the IL-3 and IL-4 signaling pathways.

Four prognostic genes were selected among the 27 targets to construct an effective prognostic model of colon cancer. Two core targets (*CCNB1* and *CCNA2*) were obtained by overlapping the PPI network targets with the four genes used in the prognostic model. *CCNB1* is an important regulator of the cell growth cycle and can affect a wide range of biological processes, including inhibition of cell proliferation, induction of cell senescence, and apoptosis [39]. *CCNB1* is widely expressed in a variety of tumors, such as ovarian cancer [40], liver cancer [41], and gastric cancer [42]. A previous study reported that *CCNB1* inhibited the activation of the p53 pathway via regulation of *FOXM1* to promote the occurrence and development of tumors [43]. Further research demonstrated that *CCNB1* was continuously downregulated after knocking out *SOX2OT,* which resulted in inhibited migration and invasion of colorectal cancer cells due to reduced expression of the mesenchymal protein N-cadherin and increased expression of the epithelial protein E-cadherin [44]. These findings suggest that *CCNB1* may play an oncogenic role in colon cancer.

Furthermore, the compound-target network of *CCNB1* revealed that multiple compounds targeted cyclin B1, including AE from DH and QR from GC. As an anthraquinone and TCM monomer, AE has a wide range of pharmacological effects, including antitumor activity [45], cardiovascular protection [46], anti-inflammatory activity [47], and immune function regulation [45]. AE antitumor activity has been reported against liver cancer [48], breast cancer [49], and gastric cancer [50]. In SW260 and HT29 colorectal cancer cells, AE treatment induced the production of reactive oxygen species and endoplasmic reticulum stress, thus inhibiting cell viability and inducing cell apoptosis [51]. QR, a natural flavonoid widely distributed in nature, possesses a variety of biological activities and exerts antioxidant, antibiosis, and antitumor effects [52]. QR treatment reportedly induced the overexpression of *TP53I3* and *RRM2* via p53 signaling, resulting in increased apoptosis and inhibiting the proliferation and metastasis of cancer cells [2]. Research has also demonstrated that QR promoted the expression of caspase-3 and increased the *BAX/BCL-2* ratio to induce apoptosis in human HT-29 colon cancer cells [53]. Taken together, these studies support the notion that AE and QR may play antitumor roles by targeting *CCNB1*.

Molecular docking simulations and molecular dynamics analysis provided further evidence supporting the predicted active compounds and target proteins, indicating that AE and QR had good binding affinities for cyclin B1. The *in vitro* results further confirmed that AE and QR inhibited the proliferation of HCT116 colon cancer cells and significantly reduced *CCNB1* expression. As *CCNB1* is known to be a tumor promoter that influences the immune activity of the hepatocellular carcinoma tumor microenvironment [54], these results suggest that AE and QR inhibit the proliferation of colon cancer cells by downregulating *CCNB1*.

## 5. Conclusions

In conclusion, this study explored the molecular targets and mechanisms of THCQF against colon cancer and constructed a relevant prognostic model using network pharmacology and bioinformatics analysis. The predicted molecular targets and active compounds of THCQF were further validated experimentally. Our findings suggest that the anticolon cancer activity of THCQF may be achieved via AE and QR targeting *CCNB1*. In addition to elucidating the novel molecular mechanism by which THCQF exerts its anticolon cancer effects, this study provides a theoretical basis for studying the anticancer mechanisms of other TCMs.

## Figures and Tables

**Table 1:** RT-PCR reaction system.

Reagent	Volume (*μ*L)
SYBR Green I (2x)	10
Primer F (10 *μ*M)	1
Primer R (10 *μ*M)	1
ddH2O	Up to 20

**Table 2:** Primer sequences using in real-time quantitative PCR.

Primer name	Primer sequence (5′-3′)
CCNB1-homo-qF	TGGTGAATGGACACCAACTCT
CCNB1-homo-qR	GCATGCTTCGATGTGGCATA

**Table 3:** Compounds in THCQF with predicted activity against colon cancer.

Mol ID	Molecule name	OB (%)	Dl	Source
MOL002281	Toralactone	46.46	0.24	DH
MOL000471	Aloe emodin	83.38	0.24	DH
MOL004912	Glabrone	52.51	0.5	GC
MOL004913	1,3-Dihydroxy-9-methoxy-6-benzofurano [3,2-c]chromenone	48.14	0.43	GC
MOL004914	1,3-Dihydroxy-8,9-dimethoxy-6-benzofurano [3,2-c]chromenone	62.9	0.53	GC
MOL004915	Eurycarpin A	43.28	0.37	GC
MOL004941	(2R)-7-hydroxy-2-(4-hydroxyphenyl)chroman-4-one	71.12	0.18	GC
MOL004949	Isolicoflavonol	45.17	0.42	GC
MOL004957	HMO	38.37	0.21	GC
MOL004959	1-Methoxyphaseollidin	69.98	0.64	GC
MOL004966	3′-Hydroxy-4′-O-methylglabridin	43.71	0.57	GC
MOL000497	Licochalcone a	40.79	0.29	GC
MOL004974	Licochalcone a	46.16	0.57	GC
MOL004978	2-[(3R)-8,8-Dimethyl-3,4-dihydro-2h-pyrano [6,5-f]chromen-3-yl]-5-methoxyphenol	36.21	0.52	GC
MOL004990	7,2′,4′-Trihydroxy-5-methoxy-3－arylcoumarin	83.71	0.27	GC
MOL004991	7-Acetoxy-2-methylisoflavone	38.92	0.26	GC
MOL000500	Vestitol	74.66	0.21	GC
MOL005000	Gancaonin G	60.44	0.39	GC
MOL005001	Gancaonin H	50.1	0.78	GC
MOL005003	Licoagrocarpin	58.81	0.58	GC
MOL005007	Glyasperin M	72.67	0.59	GC
MOL005008	Glycyrrhiza flavonol A	41.28	0.6	GC
MOL005012	Licoagroisoflavone	57.28	0.49	GC
MOL005016	Odoratin	49.95	0.3	GC
MOL005017	Phaseol	78.77	0.58	GC
MOL005020	Dehydroglyasperin C	53.82	0.37	GC
MOL000098	Quercetin	46.43	0.28	GC
MOL000359	Sitosterol	36.91	0.75	GZ
MOL001323	Sitosterol alpha1	43.28	0.78	TR
MOL001349	4a-Formyl-7alpha-hydroxy-1-methyl-8-methylidene-4aalpha, 4bbeta-gibbane-1alpha,10beta-dicarboxylic acid	88.6	0.46	TR
MOL001351	Gibberellin A44	101.61	0.54	TR
MOL000296	Hederagenin	36.91	0.75	TR
Na2so4.10h2o	Na_2_SO_4_.10H2O	NA	NA	MX

**Table 4:** Molecular docking results of compounds in THCQF with predicted activity against colon cancer.

Targets	Mol ID	Compound	Docking score
CCNB1	MOL000471	Aloe emodin	−6.124
CCNB1	MOL000098	Quercetin	−5.875
CCNA2	MOL000497	Licochalcone a	−5.181
CCNA2	MOL004912	Glabrone	−4.903
CCNA2	MOL004913	1,3-Dihydroxy-9-methoxy-6-benzofurano[3,2-c]chromenone	−5.036
CCNA2	MOL004915	Eurycarpin A	−4.934
CCNA2	MOL004949	Isolicoflavonol	−5.093
CCNA2	MOL004957	HMO	−5.146
CCNA2	MOL004959	1-Methoxyphaseollidin	−4.627
CCNA2	MOL004966	32032-Hydroxy-4′-O-Methylglabridin	−3.929
CCNA2	MOL004974	3′-Methoxyglabridin	−4.511
CCNA2	MOL004978	2-[(3R)-8,8-Dimethyl-3,4-dihydro-2h-pyrano [6,5-f]chromen-3-yl]-5-Methoxyphenol	−5.222
CCNA2	MOL005000	Gancaonin G	−4.393
CCNA2	MOL005001	Gancaonin H	−4.108
CCNA2	MOL005003	Licoagrocarpin	−5.173
CCNA2	MOL005007	Glyasperin M	−4.856
CCNA2	MOL005008	Glycyrrhiza flavonol A	−4.542
CCNA2	MOL005012	Licoagroisoflavone	−4.445
CCNA2	MOL005016	Odoratin	−4.209
CCNA2	MOL005017	Phaseol	−4.698
CCNA2	MOL005020	Dehydroglyasperin C	−5.628
CCNA2	MOL000500	Vestitol	−5.252

## Data Availability

The data supporting the findings of this study are available within the article and from TCGA and GEO databases.

## Abbreviations

AE: Aloe emodin
AUC: Area under the curve
BATMAN-TCM: Bioinformatics Analysis Tool for Molecular Mechanism of Traditional Chinese Medicine
DEG: Differentially expressed gene
DH: Dahuang
GC: Gancao
GEO: Gene expression omnibus
GO: Gene ontology
GZ: Guizhi
IC_50_: Half-maximal inhibitory concentration
IL: Interleukin
KM: Kaplan–Meier
KEGG: Kyoto Encyclopedia of Genes and Genomes
LASSO: Least Absolute Shrinkage and Selection Operator
MX: Mangxiao
PPI: Protein-protein interaction
QR: Quercetin
RMSD: Root-mean-square deviation
RMSF: Root-mean-square fluctuation
RT-PCR: Real-time polymerase chain reaction
TBST: Tris-buffered saline with Tween 20
TCGA: The Cancer Genome Atlas
TCM: Traditional Chinese medicine
TCMSP: Traditional Chinese medicine systems pharmacology
THCQF: Tao-He-Cheng-Qi formula
TR: Taoren.
